# Antimicrobial Use in COVID-19 Patients in the First Phase of the SARS-CoV-2 Pandemic: A Scoping Review

**DOI:** 10.3390/antibiotics10060745

**Published:** 2021-06-19

**Authors:** Wenjuan Cong, Ak Narayan Poudel, Nour Alhusein, Hexing Wang, Guiqing Yao, Helen Lambert

**Affiliations:** 1 Department of Population Health Sciences, Bristol Medical School, University of Bristol, Bristol BS8 2PS, UK; nour.alhusein@bristol.ac.uk (N.A.); h.lambert@bristol.ac.uk (H.L.); 2 Department of Health Sciences, University of Leicester, Leicester LE1 7RH, UK; anp13@leicester.ac.uk (A.N.P.); gy38@leicester.ac.uk (G.Y.); 3 Key Laboratory of Public Health Safety of Ministry of Education, School of Public Health, Fudan University, Shanghai 200032, China; wanghexing@fudan.edu.cn

**Keywords:** COVID-19 patients, disease severity, antibiotic use, clinical justification, secondary infections

## Abstract

This scoping review provides new evidence on the prevalence and patterns of global antimicrobial use in the treatment of COVID-19 patients; identifies the most commonly used antibiotics and clinical scenarios associated with antibiotic prescribing in the first phase of the pandemic; and explores the impact of documented antibiotic prescribing on treatment outcomes in COVID-19 patients. The review complies with PRISMA guidelines for Scoping Reviews and the protocol is registered with the Open Science Framework. In the first six months of the pandemic, there was a similar mean antibiotic prescribing rate between patients with severe or critical illness (75.4%) and patients with mild or moderate illness (75.1%). The proportion of patients prescribed antibiotics without clinical justification was 51.5% vs. 41.9% for patients with mild or moderate illness and those with severe or critical illness. Comparison of patients who were provided antibiotics with a clinical justification with those who were given antibiotics without clinical justification showed lower mortality rates (9.5% vs. 13.1%), higher discharge rates (80.9% vs. 69.3%), and shorter length of hospital stay (9.3 days vs. 12.2 days). In the first 6 months of the pandemic, antibiotics were prescribed for COVID-19 patients regardless of severity of illness. A large proportion of antibiotic prescribing for mild and moderate COVID-19 patients did not have clinical evidence of a bacterial co-infection. Antibiotics may not be beneficial to COVID-19 patients without clinical evidence of a bacterial co-infection.

## 1. Introduction

Antimicrobial resistance (AMR) kills an estimated 700,000 people every year [1]. Without intervention, the current trajectory predicts a gloomy figure of 10 million fatalities by 2050 [2]. The SARS-CoV-2 pandemic foreshadows the crisis of living with an infectious disease for which there is no treatment and the damaging consequences to our health systems and economies. At the beginning of the pandemic, with the panic of facing the unknown, many existing medicines were repurposed to treat the virus. This included widespread use of antibiotics in treatment [3,4,5,6,7]. For example, in a multi-hospital cohort study in the USA, 56.6% of 1705 patients were prescribed early empiric antibacterial therapy, of which only 3.5% were confirmed to have bacterial infection [5]. Two systematic reviews found that, of the patients reported in the included studies, 72.0% received antibiotics, and 14.3% suffered a secondary bacterial infection [4,7]. The low proportion of COVID-19 patients having co-infection or secondary infection in these studies is consistent with other findings. For example, in Italy, from the 16,654 patients who died of COVID-19, only 11% were reported to have a secondary bacterial infection (data as of 9 April 2020) [8]. In Spain, of 989 consecutive patients with COVID-19, only 72 (7.3%) had confirmed bacterial infections [9].

Overall, the pandemic may be accelerating the threat of AMR due to the increased use of antibiotics, increased exposure to hospital environments and invasive procedures used in COVID-19 treatment, while evidence for the benefits of antimicrobial use in such patients is limited. Many AMR experts have raised their concerns around the safety of using antibiotics in COVID-19 patients and called for strengthening antimicrobial stewardship (AMS) programs in the time of COVID-19 [8,10,11,12,13,14]. For example, the increased use of empirical antibiotics treatment increases the risks of *Clostridioides difficile* infection in COVID-19 patients and the emergence of multidrug resistant organism [15,16].

Guidelines have started to emerge around the use of antimicrobials in COVID-19 patients. For example, WHO guidelines recommend no antibiotic therapy or prophylaxis for patients with mild or moderate COVID-19 unless signs and symptoms of a bacterial infection exist. For severe COVID-19, a daily assessment for de-escalation of antimicrobial treatment is recommended. For elderly patients and children under five with moderate COVID-19, WHO recommends use of antibiotics categorized in the WHO access list of medicines such as co-amoxicillin [10,17].The March 2021 UK National Institute for Health and Care Excellence (NICE) rapid guideline on managing COVID-19 provides the following consensus recommendations: (1) do not use antibiotics for preventing or treating COVID-19; (2) only use antibiotics if there is a strong clinical suspicion of additional bacterial infection [18]. Other guidelines such as those of the Dutch Working Party on Antibiotic Policy [16] and the Scottish Antimicrobial Prescribing Group [19] both advise avoiding routine antibiotic use in suspected COVID-19, the importance of obtaining sputum and blood samples as well as urinary antigen testing upon admission, and a cautious antibiotic treatment of short duration of five days in patients of COVID-19 when there is a clinical suspicion of secondary bacterial infection.

There is an urgent need for further research and guidance in this field, from producing evidence-based guidelines [16], reassessing biomarkers for antimicrobial stewardship in COVID-19 patients [13], understanding drivers, benefits, and disbenefits of antibiotic use, and assessing the wider impact of the pandemic on antimicrobial use (AMU) and AMR. In this scoping review, we aim to: add to the research evidence on prevalence and patterns of antimicrobial use in the treatment of COVID-19 patients; identify the most commonly used antibiotics and clinical scenarios associated with AMU; and to explore any impact of AMU on patient treatment outcomes.

## 2. Results

### 2.1. Study Selection

**Figure 1:**
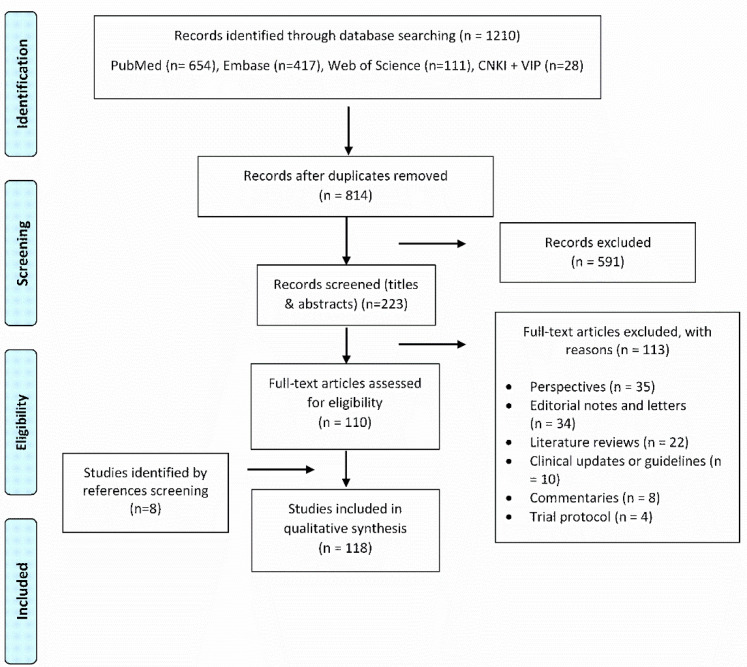
Prisma chart.

A total of 1216 records were identified through database searching. After duplicates were removed and irrelevant records of COVID-19 research not related to clinical treatment of COVID-19 patients were excluded, 223 records were screened for eligibility. A further 113 studies that reported neither antimicrobial use in treatment nor patients with co-infections were excluded. The remaining 110 articles and an additional eight articles that were identified by searching the reference lists of the retrieved articles and the authors’ reference collections, led to a total of 118 full-text articles being included for review (Figure 1).

### 2.2. Description of Included Studies

Of 118 included studies, 59 were case series or case reports, 47 were observational studies (all types of observational study except cohort studies), seven were randomized controlled trials, and five were cohort studies. Most of the studies were conducted in low- and middle-income countries, consonant with the trajectory of the pandemic at that stage, with the majority conducted in China (51.7%), followed by USA Italy and France (Supplementary Table S1). Highest number of studies were from East Asia and Pacific (55.9%), followed by Europe and Central Asia (22.0%) and North America (14.4%). All the studies reported data of hospitalized COVID-19 patients. There were no reports of non-hospitalized COVID-19 patients in our results.

### 2.3. Antibiotic Prescribing and Illness Severity

Severity of illness was not reported in all studies. Just over half reported the severity of illness using four categories (severe, critical, moderate, and mild) and the rest remainder used three groups (severe, moderate, and mild). In order to explore the potential role of severity of illness in decisions regarding antibiotic prescribing, we grouped severity of illness into two broader categories: severe or critical, and moderate or mild. A total of 2630 patients (41.9%) fell into the severe or critical group and 3649 patients (58.1%) into mild or moderate group.

In the included studies, 8501 out of 10,329 COVID-19 patients (82.3%) were prescribed antibiotics. There was little difference in the mean rates of antibiotic prescribing with 75.4% in severe or critical vs. 75.1% in mild or moderate groups (Table 1).

### 2.4. Antibiotic Prescribing and Health Outcomes

We further explored the relationship between antibiotic prescribing and health outcomes (length of hospital stay (LOS), discharge rate, and mortality rate (all these health outcomes were calculated at the time of publication of those studies; some patients were still in hospital and these patients were not included in their calculation). The results show that patient mortality was higher in studies for patients all given antibiotics compared to studies that majority of patients were not given antibiotics (26.5% vs. 2.3%), LOS was longer (12.5 days vs. 10.3 days), and discharge rate was also higher (76.2% vs. 73.2%) (Table 2).

### 2.5. Frequently Prescribed Antibiotics

We extracted the details of prescribed antibiotics from all the included studies where this information was available. Further, 33.9% of included studies did not report the details of antibiotics but only mentioned that antibiotics or empirical antibiotics were used in treatment.

**Figure 2:**
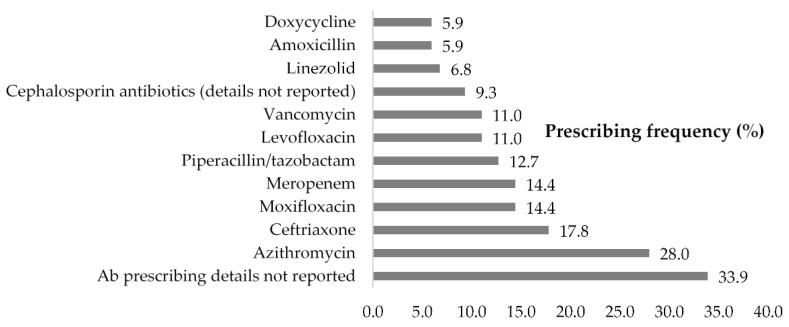
Frequently prescribed antibiotics for hospitalized COVID-19 patients.

Among the 78 studies that reported type of antibiotics used in the treatment of COVID-19 patients, (Figure 2) azithromycin (macrolides and ketolides) was the most frequently prescribed antibiotic (accounting for 28.0% of studies); followed by ceftriaxone (17.8%), moxifloxacin (14.4%), meropenem (14.4%), and Piperacillin/tazobactam (12.7%). It is not possible to tabulate prescribing percentages of these frequently prescribed antibiotics for the treatment of hospitalized COVID-19 patients as most studies, except case report or case series, did not report the percentage of each prescribed antibiotic in the treatment of COVID-19 patients. Notably, the frequently prescribed antibiotics are all broad-spectrum antibiotics.

### 2.6. Antibiotic Prescribing Scenarios

We summarized 20 different scenarios when antibiotics were prescribed based on the evidence available in our included studies (Table 3). We asked two experienced clinicians in infectious diseases with expertise in AMS practices to classify each antibiotic prescribing scenario as: (1) with clinical justifications; (2) without clinical justifications; (3) not sure. In addition to microbiological analysis; sepsis, elevated white blood cells or procalcitonin are also signs of bacterial infection and antibiotics prescribed under those circumstances were considered as “with clinical justifications”. There were some ambiguous cases (around 30% of scenarios) on which both experts found difficult to make judgements regarding whether antibiotics should be prescribed or not; we categorized those as “not sure”. A relatively high proportion (around 45%) of scenarios described in the included studies were categorized by both experts as “without clinical justifications”.

We also categorized the frequency of each antibiotic prescribing scenario by illness severity (Supplementary Table S2). We found that only 12.9% of severe or critically ill patients were prescribed antibiotics with clinical justifications, and this proportion was 13.6% for mild or moderate patients. The proportion of patients prescribed antibiotics without clinical justification was 51.5% vs. 41.9% for patients with mild or moderate illness and those with severe or critical illness.

### 2.7. Severity of Illness, Antibiotic Prescribing Justifications, and Health Outcomes

All studies with all severe or critical patients had a higher mortality rate than all studies with mild or moderate patients (53.1% vs. 0.2%), similarly with lower discharge rate (96.2% vs. 36.6%) and higher LOS (17.4% vs. 8.7%) (Table 4).

Mortality rate was lower for those patients who were provided antibiotics with clinical evidence of infections compared to those who were given without clinical justifications (9.5% vs. 13.1%), discharge rate was higher (80.9% vs. 69.3%) and LOS was lower (9.3 days vs. 12.2 days) (Table 5).

### 2.8. Secondary Infections and Health Outcomes

Nine of the 118 studies reported on secondary infections. Out of a total sample size of 820 in these studies, 74.4% patients had diagnosed secondary infections (*n* = 610) and 51.3% of these patients were serious or critically ill (*n* = 313). In perspective to our total sample size across all studies (6279 patients), the percentage of patients with confirmed or diagnosed secondary infections was 9.7%. Compared to total patients (*n* = 6279), patients with secondary infections had higher LOS (20.4 days vs. 12.4 days), lower discharge rate (54.8% vs. 65.6%), and higher mortality rate (43.7% vs. 16.3%) (Table 6).

### 2.9. Gender and Health Outcomes

We found that male patients compared to female patients had a higher mortality rate (37.7% vs. 20.0%), lower discharge rate (75.2% vs. 91.7%), and longer LOS (13.4 days vs. 11.4 days) (Supplementary Table S3). These findings are in line with other recent studies conducted with COVID-19 patients across the world [20,21].

### 2.10. Study Design and Country Economic Status

We conducted a stratified analysis of antibiotic prescribing rate, length of hospital stay, discharge rate, and mortality rate by study design and incomes of the countries where studies originated. There was little variation in antibiotic prescribing rate, length of hospital stay, and discharge rate by study design. The highest mortality rate was reported in cohort studies (25%) and lowest reported in randomized control trials (3%) (Supplementary Table S4). Reported antibiotic prescribing rates, length of stay, and mortality rates were also similar between low-and-middle income countries (LMICs) and high-income countries (HICs) based on World Bank Classfication. However, discharge rates were considerably lower in LMICs (60.2%) compared to HICs (81.9%) (Supplementary Table S5).

## 3. Discussion

As the numbers of people with COVID-19 continue to increase globally, the widespread use of antibiotics for the treatment of COVID-19 patients and the potential consequences of this for AMR are a growing concern.

Our included studies provide data on patients in hospital settings; this was not an inclusion criterion but none of the studies identified presented data on non-hospitalized patients (e.g., care home patients, patients in the community, or treated as outpatients). This is probably because data outside hospital settings are harder to obtain and were not explored in the first six months of the pandemic. Data synthesis from the 118 studies included in this review shows that around 82.3% of hospitalized COVID-19 patients were prescribed antibiotics, whereas antibiotic prescribing percentages of COVID-19 patients were almost 100% in the early clinical reports from hospitals in Wuhan [22,23,24]. It is unsurprising that doctors were giving antibiotics almost universally to treat this previously unknown respiratory infection at the beginning of the pandemic; resort to antibiotics would be expected to decline with increasing knowledge about the novel coronavirus. More surprisingly, the antibiotic prescribing rate does not vary with illness severity (75.4% in severe or critical vs. 75.1% in mild or moderate patients).

Antibiotics use may be warranted in managing COVID-19 patients with suspected bacterial co-infections and severe/critically ill patients with increased risks of developing bacterial co-infections due to long hospital stays or immunosuppression [18]. Differentiating bacterial secondary co-infection from severe COVID-19 inflammatory reaction is clinically difficult, and commencing empirical antibiotic therapy in these circumstances is understandable. COVID-19-related concerns or unknowns may change prescription behaviors of healthcare professionals (HCPs) and drive antibiotic use [25]. Additionally, diagnostic confirmation of secondary bacterial/fungal infections through microbiological culturing is costly, time consuming, and not available in smaller hospitals, making rapid exclusion of secondary infections very difficult and pushing HCPs to err on the side of treating with antibiotics both as directed and empirical therapy.

Previous experience of viral respiratory infection may also have played a role; for example, in the USA, 30% of critical ill patients had bacterial co-infections in the 2009 influenza A (H1N1) pandemic [26], while around 23% of severe influenza patients had bacterial co-infections in the 2013–2014 flu season [27]. In contrast, evidence reviewed by NICE up to March 2021 [18] suggests that less than 8% of people with COVID-19, and as few as 0.1% of hospitalized patients, have bacterial co-infections, but it is understandable that rates similar to those seen in influenza may have been assumed initially in COVID-19 patients.

However, the high rates of antibiotic prescribing for mild or moderate COVID-19 patients (around 51.5% of whom were given antibiotics suggests more indiscriminate use in the absence of clinical evidence for possible bacterial co-infection. Antibiotic prescribing for mild and moderate COVID-19 patients is inconsistent with WHO COVID [17] and recently updated UK NICE guidelines [18] and not only indicates the emerging challenges of antimicrobial stewardship practice in hospitals around the world, but suggests a potential increase in resistant bacterial pathogens in affected countries.

Moreover, the antimicrobials found to have been most frequently prescribed in this review (azithromycin, ceftriaxone, moxifloxacin, meropenem, piperacillin/tazobactam) are all classified as critically important antimicrobials (CIA) for human medicine [28]. Persistent use of these critically important antibiotics will provoke the emergence of MDR strains and a decline in the effectiveness of these compounds [29], posing a threat to survival rates from serious infections, neonatal sepsis, and hospital infections, thus limiting the potential health benefits of surgery, transplants, and cancer treatment [30]. A systematic review has provided evidence on the emergence of AMR after mass azithromycin distribution for trachoma control programmes in Sub-Saharan Africa; macrolide resistance after azithromycin distribution was reported in three of the five organisms studied and there was little evidence for absence of resistance in Chlamydia trachomatis after azithromycin treatment, suggesting that azithromycin may not remain effective for future trachoma programmes [31]. The impact of widespread use of ceftriaxone on development of resistance to third generation cephalosporins among clinical strains of Enterobacteriaceae and other non-enteric bacteria is already well known [32,33,34]. If resistance to azithromycin, ceftriaxone, and other broad-spectrum antibiotics becomes widespread due to their massive use during the pandemic, there would be very few alternative antibiotics available in the market and these alternatives antibiotics are likely to be unaffordable for the majority of patients, especially in low and middle-incomes countries.

The low proportion of antibiotic prescribing (less than 14%) for COVID-19 patients with a clinical justification for assuming the presence of bacterial infection provides further evidence that clinical indication was not the primary driver of antibiotic therapy. Overall, 40–50% of antibiotic prescribing (41.9% for severe or critical patients vs. 51.5% for mild or moderate patients) in the included studies occurred without clinical indications of bacterial infection. Around 45% of antibiotic prescribing scenarios described in the reviewed studies were classified as “not sure” for severe or critical patients, but only around 35% for mild or moderate patients, suggesting that in cases of greater severity, HCPs are more likely to resort to antibiotic treatment even in the absence of clinical indications suggestive of bacterial infection. Only 9.7% of all COVID-19 patients with documented severity status in the reviewed studies were reported to have a secondary bacterial infection, consistent with findings from other publications [3,4]. Among the three most common prescribing scenarios reported in our included studies (Supplementary Table S3), two involved unwarranted antibiotic prescribing.

Our analysis also provides evidence regarding the effect of antibiotic use on treatment outcomes, as measured by LOS, discharge rate, and mortality rate of hospitalized COVID-19 patients. We found that overall, patients who were provided antibiotics with a clinical justification compared with those who were given antibiotics without clinical justification showed lower mortality rates (9.5% vs. 13.1%), higher discharge rates (80.9% vs. 69.3%), and shorter length of hospital of stay (9.3 days vs. 12.2 days). This evidence supports and strengthens the rationale for current guidance that COVID-19 patients without a confirmed or suspected (based on clinical/microbiological indicators) secondary infection should not be prescribed antibiotics. This is consonant with findings from a retrospective study examining treatment outcomes in 1123 COVID-19 patients from Wuhan that compared antibiotics treatment between patients with suspected bacterial infection compared to those with no evidence of bacterial infection. Antibiotic therapy including penicillin and meropenem treatment was found to be associated with increased mortality in patients with no evidence of bacterial infection and the authors concluded that most patients without suspected bacterial infection would not benefit from antibiotics treatment [35]. A retrospective study that reviewed the medical charts of 48 intubated ICU patients admitted between April and May 2020 in Switzerland, also reported that early administered antibiotics do not appear to significantly impact mortality or delay hospital-acquired infections in critically ill COVID-19 patients [36].

Although empirical, adjuvant, and prophylactic use of antibiotics for severe and critical patients is still endorsed in WHO and China guidelines [17,37], this may not confer the expected benefits. Our results suggest that antibiotic treatment may not improve COVID-19 patients’ treatment outcomes. Patients who were prescribed antibiotics with suspected bacterial infection were also more likely to have negative clinical outcomes, including higher LOS (20.4 days vs. 12.4 days), lower discharge rate (54.8% vs. 65.6%), and higher mortality rate (43.7% vs. 16.3%) compared to average patient treatment outcomes. Large multi-center studies are urgently needed to provide direct evidence for our findings and further investigate the impact of antibiotic treatment on mortality and other treatment outcomes of COVID-19 patients with different illness severities.

Strengths of this review include coverage of 118 studies across a wide range of study designs published during the six-month review period, giving a full landscape of the prevalence and patterns of antibiotic prescribing for COVID-19 patients in hospital settings during the first wave of the pandemic. Although more is now known about COVID-19 and effective treatments, as it spreads across the globe, there is no reason to believe that antibiotics are not still being used widely, particularly in more recently affected low and middle-income countries with limited resources, so our findings continue to have relevance. The review also has several inevitable limitations. First, we relied on published online research article searching in selected databases; this may result in publication bias. Second, we excluded studies not available in English or Chinese, which may cause language bias as we were not able to include studies from European countries that were published in local languages. Third, the severity classifications given in the included papers varied between studies: just over half reported severity of illness using four categories (critical, severe, moderate, and mild) and the remainder used three groups (severe, moderate, and mild). This necessitated merging patients with reported illness severity into just two groups (severe or critical, and moderate or mild) in our analysis. Our findings concerning relationships between illness severity, antibiotic prescribing, and treatment outcomes should therefore be treated with caution. Finally, we did not assess the quality of the included studies and were unable to conduct meta-analysis due to the heterogeneity of the studies under review. This may result in less robust and generalizable findings. However, the inclusion of a wide range of study designs helps to provide a more complete picture of global antibiotic prescribing patterns during the COVID-19 pandemic than is available in the current scientific literature.

## 4. Materials and Methods

This rapid review was undertaken to identify, synthesize, and analyze findings from studies that reported antibiotic use in the treatment of COVID-19 patients. The review was conducted to comply with PRISMA guidelines for Scoping Reviews (http://www.prisma-statement.org/Extensions/ScopingReviews accessed on 9 June 2020) [38]; and the protocol was registered with the Open Science Framework (OSF): http://osf.io/vp6t5 (accessed on 23 July 2020). We selected a scoping rather than systematic review approach in order to maximize data inclusion from a wide range of study types.

### 4.1. Search Strategy

The following databases: Web of Science, EMBASE, PubMed, and two Chinese academic databases (CNKI and VIP) were searched to identify relevant studies from 1 Dec 2019 up to 15 June 2020; no limits were set on the country where study was conducted, and we excluded any studies not available in English or Chinese. The search terms were: ((“COVID-19” or “SARS-CoV-2” or “Coronavirus disease 2019” or “severe acute respiratory syndrome coronavirus-2”) and ((“antibiotic prescribing” or “antibiotic use” or “antibiotic*”) or “antimicrobial *” or “antimicrobial therapy” or “antimicrobial resistance” or “antimicrobial stewardship”)).

#### 4.1.1. Web of Science

All Fields = (COVID-19 and antibiotic*) or (SARS-CoV-2 and antibiotic*) or (Coronavirus disease 2019 and antibiotic*) or (severe acute respiratory syndrome coronavirus-2 and antibiotic*)

All Fields = (COVID-19 and antimicrobial*) or (SARS-CoV-2 and antimicrobial*) or (Coronavirus disease 2019 and antimicrobial*) or (severe acute respiratory syndrome coronavirus-2 and antimicrobial*)

#### 4.1.2. PubMed

(COVID-19 or SARS-CoV-2 or Coronavirus disease 2019 or severe acute respiratory syndrome coronavirus-2) and antimicrobial*

(COVID-19 or SARS-CoV-2 or Coronavirus disease 2019 or severe acute respiratory syndrome coronavirus-2) and antibiotic*

#### 4.1.3. Embase

(antibiotic* or antibiotic prescribing or antimicrobial resistance or antibacterial*) and (severe acute respiratory syndrome coronavirus-2 or COVID-19 or SARS-CoV-2 or Coronavirus disease 2019).

#### 4.1.4. CNKI and VIP

(COVID-19 or SARS-CoV-2 or Coronavirus disease 2019 or severe acute respiratory syn-drome coronavirus-2) and (antibiotic* or antibiotic use or antimicrobial resistance or antibacterial*)

### 4.2. Inclusion, Exclusion Criteria, and Study Selection Process

Articles fulfilling the following criteria were considered for inclusion in the review. Full-text articles only were included.

#### 4.2.1. Inclusion criteria

1. All types of clinical studies (randomized control trial (RCT), cohort, case report including case series, other observational studies (except cohort)) about the use of antibiotics to treat patients with COVID-19.
2. Studies reporting patients diagnosed with COVID-19 and receiving antibiotic treatment, without restrictions on age, race, gender, geographical location.
3. Studies which had mentioned antibiotic treatment and also reporting treatment outcomes.
4. Studies reporting COVID-19 patients with bacterial co-infections.

#### 4.2.2. Exclusion Criteria

1. Animal studies, in vitro experiments, in silico screening/drug modeling, molecular mechanism, and other aspects of COVID-19 research where not related to or mentioned antibiotic use (ABU).
2. Conference abstracts.
3. Commentaries and editorial letters not reporting ABU.
4. Literature review not reporting ABU.
5. Trial protocol.
6. Case report and case series not reporting ABU.
7. Full-text articles not available in English or Chinese.
8. Studies reporting suspected or asymptomatic COVID-19 patients.
9. Studies reporting COVID- 19 patients in primary care settings such as GP and community health center.

Titles and abstracts were screened initially, and full texts were retrieved of articles which appeared to fulfil the inclusion criteria. Two independent, duplicate screenings were undertaken by WC and NA for all search results. Disagreement was resolved by consensus. Additional studies were identified by searching the reference lists of retrieved articles and the authors’ reference collections.

### 4.3. Data Extraction

A bespoke data extraction form was developed and validated through two independent, duplicate extraction of data from five relevant studies. Data extraction for antibiotic use in the treatment of COVID-19 patients included: publication details, region, type of COVID-19 patients, age, gender, number of patients reported, study type (case report including case series, RCT, cohort, other observational studies), type of patients (mild or moderate, severe, or critical), antimicrobial prescribing rate (overall and for different types of patient, details of antibiotics prescribed if reported, antibiotic prescribing scenarios (under which circumstance antibiotic was prescribed), whether antibiotic treatment had complied with AMS practice (yes, no, or not sure), mean length of hospital stay, discharge rate, and mortality rate.

Data extraction for COVID-19 patients with secondary bacteria or fungal infections included: publication details, region, gender, reported number of patients with co-infections, type of patients with co-infections (severe or critical ill), type of co-infections, details of antibiotic used for the treatment of co-infections, length of stay, discharge rate, mortality rate of patients with co-infections, gender, age, and underlying health conditions of patient with co-infections if reported.

### 4.4. Data Synthesis and Analysis

We looked at the overall antibiotic prescribing rate, scenarios of antibiotics prescribing, types of antibiotic use, and health outcomes of patients. In this review, data extraction and analysis were performed in Microsoft Excel. We conducted descriptive synthesis, taking into account of the sample size of each study (i.e., weighted mean) while calculating pooled estimates of outcome variables (i.e., antibiotics prescribing%, mortality rate, discharge rate, length of stay). We also conducted subgroup analyses of COVID-19 patients who were given antibiotics during hospital admission, most frequently used antibiotics for these patients, mean percentage of clinically justifiable antibiotic use, most frequently used medicines (other than antibiotics) for COVID-19 patients, proportion of COVID-19 patients having underlying health conditions, mean length of stay in hospital, mean discharge rate, and mean mortality rate. In addition, we also conducted stratified analysis of major outcome variables (antibiotic prescribing rate, length of hospital stay, discharge rate, and mortality rate) by type of study design and income of originating country. The studies were divided into two major income groups (High-Income Countries and Low-and-Middle Income Countries) using World Bank classification [39]. We also classified the region of origin of all included studies using the World Bank classification into seven groups, as reported in the Supplementary Table S1.

As described above, we also investigated proportion of patients experiencing co-infections (fungal, bacterial, or other), most frequently used antibiotics for the treatment of co-infections, mean length of stay for patients with co-infections, mean discharge rate and mean mortality rate for the patients with co-infections, and proportion of patients with co-infection having underlying health conditions. We present these results by age and gender of patients.

## 5. Conclusions

This review and evidence synthesis suggest that during the first six months of the COVID-19 pandemic, antibiotic prescribing in hospitals was not associated with illness severity. A large proportion (40–50%) of antibiotic prescribing for COVID-19 patients did not have clinical indications of a bacterial co-infection; around half of COVID-19 patients with mild or moderate illness, had been prescribed antibiotics in the reports and studies we reviewed. Patients without clinical evidence of a bacterial co-infection should not receive antibiotics treatment according to international guidelines.

The evidence reviewed suggests that where secondary bacterial infection is absent, antibiotic prescribing may not be beneficial to treatment outcomes for COVID-19 patients. Until more clinical data become available to verify these findings, considerable caution is warranted when considering antibiotic treatment in COVID-19 cases, even for severe and critically ill patients. The widespread use of antibiotics for COVID-19 may not only magnify the problem of antibiotic resistance globally and render currently available antibiotics ineffective, but also provide little or no benefit for COVID-19 patients. A further scoping review to capture changes in global prevalence and patterns of antibiotic prescribing for COVID-19 patients in hospital settings from June 2020 to Aug 2021 is currently underway.

## Figures and Tables

**Table 1:** Severity of illness and antibiotic prescribing.

Illness Severity of COVID-19 Patients	Patient Size *n* (%)	Mean Antibiotic Prescribing (%)
Severe and critical patients	2630 (41.9)	75.4
Mild and moderate	3649 (58.1)	75.1
Total	6279 (100.0)	75.2

**Table 2:** Antibiotic prescribing categories and outcomes.

SN	Category of Antibiotic Prescribing	LOS (Mean Days)	Discharge (Mean%)	Mortality (Mean%)
1	All given abs (58 studies)	12.5	76.2	26.5
2	Majority are given abs (37 studies)	14.3	57.9	13.1
3	Majority not given abs (11 studies)	10.3	73.2	2.3

**Table 3:** Antimicrobial prescribing scenarios of hospitalized COVID-19 patients.

Antimicrobial Prescribing Scenarios with Clinical Justifications (A)	Antimicrobial Prescribing Scenarios without Clinical Justifications (B)	Antimicrobial Prescribing Scenarios not Sure whether with or Without Clinical Justifications (C)
Scenario 1: Microbiological analysis such as blood, stool, urine, or sputum culture was tested positive.	Scenario 4: Respiratory failure such as acute respiratory distress syndrome (ARDS)	Scenario 9: Patients are older or frail, or have a pre-existing comorbidity such as immunosuppression (Solid organ transplant recipients who remain on long term immune suppression therapy), HIV patients, or significant heart or lung disease (for example bronchiectasis or COPD, systemic lupus erythematosus), or have a history of severe illness following previous lung infection
Scenario 2: Pulmonary aspergillosis	Scenario 7: C reactive protein higher (around 30 mg/L, normal range 0–8 mg/L)	Scenario 10: Elder patient with other type of cancer not listed above, diabetes, hypertension
Scenario 3: Septic shock or sepsis	Scenario 8: Patients received ventilation or mechanical ventilation	Scenario 12: Azithromycin was used as a combination therapy with hydroxychloroquine
Scenario 5: Procalcitonin >0.5 ng/mL	Scenario 11: Pregnant woman for caesarean section, or with suspected bronchitis	Scenario 14: Mentioned that “Abs were used as an empirical treatment when it was very difficult to exclude bacterial co-infection”; or “abs were used if needed and this decision was based on health care providers’ discretion” or “abs were initialled at the beginning but was discontinued after COVID-19 was confirmed or after microbiological culture analysis tested negative” or “abs were empirically used and patient developed bacterial infection later (case report) or a high percentage of patients developed bacterial co-infections later
Scenario 6: a high percentage of neutrophils (neutrophilia), WBC count	Scenario 13: Antimicrobial treatment was given without any justifications (Not reporting any suspected bacterial/fungal co-infection symptoms, or any lab test results indicating possible bacterial/fungal infections)	Scenario 15: Abs were used for most patients (higher prescribing rate) to cover possible bacterial co-infections; however only a minor percentage of patients developed bacterial/fungal infections
Scenario 18: Paediatric patient (infant, preterm neonate) with abnormal blood cell test or CRP levels; or suspected sepsis etc	Scenario 16: Abs were reported to be used as an empirical/adjuvant/concomitant/standard treatment; and patients were given abs on admission, or before randomization into different trial groups for some trials)	Scenario 17: Dual or triple antibiotics used
	Scenario 19: Patient with acute appendicitis	
	Scenario 20: Patient with digestive symptoms	

**Table 4:** Severity of illness and health outcomes.

Severity of Illness (Categories)	LOS (Mean Days)	Discharge (Mean%)	Mortality (Mean%)
All severe/critical (16 studies)	17.4	36.6	53.1
Majority were severe/critical (4 studies)	18.0	77.9	5.8
Majority were mild/moderate (33 studies)	12.0	60.5	4.8
All mild/moderate (20 studies)	8.7	96.2	0.2

**Table 5:** Antibiotic prescribing justifications and health outcomes.

Antibiotic Prescribing Justified or Not	LOS (Mean Days)	Discharge (Mean%)	Mortality (Mean%)
A-with clinical justifications” (*n* = 14)	9.3	80.9	9.5
B-without clinical justifications (*n* = 49)	12.2	69.3	13.1
C-not sure (*n* = 47)	14.1	61.1	24.8

**Table 6:** Secondary infections and health outcomes.

Descriptions	Severe/Critical *n* (%)	Mild/Moderate *n* (%)	Mean Length of Stay (Days)	Mean Discharge Rate (%)	Mean Mortality Rate (%)
Total patients with secondary infections (*n* = 610)	313 (51.3%)	297 (48.7%)	20.4	54.8	43.7
Total sample size (*n* = 6279)	2630 (41.9%)	3649 (58.1%)	12.4	65.6	16.3

## Supplementary Materials

The following are available online at https://www.mdpi.com/article/10.3390/antibiotics10060745/s1, Supplementary Table S1: Description of included studies; Supplementary Table S2: Severity of illness and scenario of antibiotic prescribing; Supplementary Table S3: Types of study based on gender and health outcomes (LOS, discharge and mortality); Supplementary Table S4: Antibiotic prescribing, length of stay, discharge rate and mortality rate by study designs; Supplementary Table S5. Antibiotic prescribing, length of stay, discharge rate and mortality rate by income of the study countries. Annex 1 References list included for this review.
