# Supplementary material for: Antimicrobial Use in COVID-19 Patients in the First Phase of the SARS-CoV-2 Pandemic: A Scoping Review
Source: Antibiotics (Basel). 2021 Jun 19;10(6):745. doi: 10.3390/antibiotics10060745 (PMC8235357; doi:10.3390/antibiotics10060745)
Supplement: Supplementary file 1 [file antibiotics-10-00745-s001.zip › antibiotics-1197200-supplementary.pdf]

antibiotics

MDPI

Supplementary Material

Supplementary Table S1. Description of included studies.

SN	Description	Frequency (n)	Percentage (%)
**Types of studies**			
1	Case reports/case series	59	50·0
2	RCT	7	5·9
3	Cohort	5	4·2
4	Observational studies (except cohort studies)	47	39·8
Total		118	100
**Study countries or areas**			
1	LMICs (China, Iran, Bhutan, Brazil, Colombia, Iraq, Nepal, Philippines, Uganda, Vietnam)	72	61·0
2	HICs (USA, Italy, France, Spain, United Kingdom, Belgium, Hong Kong (China), Japan, Poland, South Korea)	46	39·0
Total		118	100
**Region of the studies**			
1	East Asia and Pacific	66	55.9
2	Europe and Central Asia	26	22.0
3	Latin America and the Caribbean	2	1.7
4	Middle East and North Africa	4	3.4
5	North America	17	14.4
6	South Asia	2	1.7
7	Sub-Saharan Africa	1	0.8
Total		118	100

Supplementary Table S2. Severity of illness and scenario of antibiotic prescribing.

SN	Scenario of antibiotics prescribing	Severe and critical illness and scenario of antibiotic prescribing		Mild and moderate illness and scenario of antibiotic prescribing	
		Frequencies (n)	Percentage (%)	Frequencies (n)	Percentage (%)
1	Scenario 1 (A)	3	4·8	2	3·0
2	Scenario 4 (B)	2	3·2	2	3·0
3	**Scenario 6 (A)**	4	6·5	6	9·1
4	Scenario 7 (B)	2	3·2	4	6·1
5	Scenario 8 (B)	2	3·2	5	7·6
6	**Scenario 9 (C)**	8	12·9	2	3·0
7	Scenario 10 (C)	2	3·2	2	3·0
8	**Scenario 12 (C)**	4	6·5	5	7·6
9	**Scenario 13 (B)**	8	12·9	13	19·7
10	**Scenario 14 (C)**	11	17·7	10	15·2
11	Scenario 15 (C)	2	3·2	2	3·0
12	**Scenario 16 (B)**	11	17·7	9	13·6
13	Scenario 17 (C)	1	1·6	2	3·0
14	Scenario 18 (A)	1	1·6	1	1·5
15	Scenario 20 (B)	1	1·6	1	1·5
	Total	62	100	66	100

[Note: every study may have more than one abs prescribing scenario; A- with clinical justifications; B – without clinical justifications; C – not sure].

Supplementary Table S3. Types of study based on gender and health outcomes (LOS, discharge and mortality) .

SN	Gender breakdown of studies	LOS (mean days)	Discharge (mean %)	Mortality (mean %)
1	Studies with female patients only (18 studies)	11·4	91·7	20·0
2	Studies with male patients only (27 studies)	13·4	75·2	37·5
3	Studies with mix of male and female patients (72 studies)	13·1	61·5	12·6

[Note: 1 study did not provide required information] .

Supplementary Table S4. Antibiotic prescribing, length of stay, discharge rate and mortality rate by study designs.

Study design	Antibiotic prescribing rate (%)	Length of hospital stay (days)	Discharge rate (%)	Mortality rate (%)
Case series and case reports (n= 59 studies)	89.3	12.5	75.6	24.1
Cohort studies (n = 5 studies)	82.1	16.7	66.6	25.0
Observational studies (except cohort studies) (n= 47)	73.2	13.3	60.1	11.0
Randomised control trials (n = 7 studies)	79.9	11.4	70.1	3.0

Supplementary Table S5. Antibiotic prescribing, length of stay, discharge rate and mortality rate by income of the study countries.

Income of the countries.	Antibiotic prescribing rate (%)	Length of hospital stay (days)	Discharge rate (%)	Mortality rate (%)
**High income countries (HICs) (n= 44 studies)**	81.1	12.0	81.9	18.1
**Low-and-middle income countries (LMICs) (n= 68 studies)**	83.3	13.6	60.2	18.3

(**Note:** We excluded two studies from HICs and four studies from LMICs from analysis because they did not report the above information).

Appendix 1 All included studies – references list

Case studies (1-59)

1. Chaumont H, Etienne P, Roze E, Couratier C, Roger PM, Lannuzel A. Acute meningoencephalitis in a patient with COVID-19. Rev Neurol (Paris). 2020;176(6):519-21.
2. Qiu H, Wander P, Bernstein D, Satapathy SK. Acute on chronic liver failure from novel severe acute respiratory syndrome coronavirus 2 (SARS-CoV-2). Liver Int. 2020;40(7):1590-3.
3. Martens T, Vande Weygaerde Y, Vermassen J, Malfait T. Acute Type A Aortic Dissection Complicated by COVID-19 Infection. Ann Thorac Surg. 2020;110(5):e421-e3.
4. Tay HS, Harwood R. Atypical presentation of COVID-19 in a frail older person. Age Ageing. 2020;49(4):523-4.
5. Spezzani V, Piunno A, Iselin HU. Benign COVID-19 in an immunocompromised cancer patient - the case of a married couple. Swiss Med Wkly. 2020;150:w20246.
6. Sise ME, Baggett MV, Shepard JO, Stevens JS, Rhee EP. Case 17-2020: A 68-Year-Old Man with Covid-19 and Acute Kidney Injury. N Engl J Med. 2020;382(22):2147-56.

7. Xu Man ZH, Niu Xiaoguang. A case of novel coronavirus pneumonia first diagnosed in ophthalmology with acute meibomitis and subconjunctival hemorrhage. Chinese Journal of Experimental Ophthalmology. 2020;38(04):2.
8. Han P, Li F, Cao P, Hu S, Kong K, Deng Y, et al. A case report with COVID-19 during perioperative period of lobectomy. Medicine (Baltimore). 2020;99(22):e20166.
9. LeVine S, Dhakal GP, Penjor T, Chuki P, Namgyal K, Tshokey, et al. Case Report: The First Case of COVID-19 in Bhutan. Am J Trop Med Hyg. 2020;102(6):1205-7.
10. Fang X, Zhao M, Li S, Yang L, Wu B. Changes of CT findings in a 2019 novel coronavirus (2019-nCoV) pneumonia patient. Qjm. 2020;113(4):271-2.
11. Liao X, Yang H, Kong J, Yang H. Chest CT Findings in a Pregnant Patient with 2019 Novel Coronavirus Disease. Balkan Med J. 2020;37(4):226-8.
12. Lescure FX, Bouadma L, Nguyen D, Parisey M, Wicky PH, Behillil S, et al. Clinical and virological data of the first cases of COVID-19 in Europe: a case series. Lancet Infect Dis. 2020;20(6):697-706.
13. Huang Q, Deng X, Li Y, Sun X, Chen Q, Xie M, et al. Clinical characteristics and drug therapies in patients with the common-type coronavirus disease 2019 in Hunan, China. Int J Clin Pharm. 2020;42(3):837-45.
14. Wang Z, Chen X, Lu Y, Chen F, Zhang W. Clinical characteristics and therapeutic procedure for four cases with 2019 novel coronavirus pneumonia receiving combined Chinese and Western medicine treatment. Biosci Trends. 2020;14(1):64-8.
15. Zhu L, Wang J, Huang R, Liu L, Zhao H, Wu C, et al. Clinical characteristics of a case series of children with coronavirus disease 2019. Pediatr Pulmonol. 2020;55(6):1430-2.
16. Ding Q, Lu P, Fan Y, Xia Y, Liu M. The clinical characteristics of pneumonia patients coinfected with 2019 novel coronavirus and influenza virus in Wuhan, China. J Med Virol. 2020;92(9):1549-55.
17. Lu T, Pu H. Computed Tomography Manifestations of 5 Cases of the Novel Coronavirus Disease 2019 (COVID-19) Pneumonia From Patients Outside Wuhan. J Thorac Imaging. 2020;35(3):W90-w3.
18. Piersigilli F, Carkeek K, Hocq C, van Grambezen B, Hubinont C, Chatzis O, et al. COVID-19 in a 26-week preterm neonate. Lancet Child Adolesc Health. 2020;4(6):476-8.
19. Wang J, Li X, Cao G, Wu X, Wang Z, Yan T. COVID-19 in a Kidney Transplant Patient. Eur Urol. 2020;77(6):769-70.
20. Leonetti A, Facchinetti F, Zielli T, Brianti E, Tiseo M. COVID-19 in lung cancer patients receiving ALK/ROS1 inhibitors. Eur J Cancer. 2020;132:122-4.
21. Morlacchi LC, Rossetti V, Gigli L, Amati F, Rosso L, Aliberti S, et al. COVID-19 in lung transplant recipients: A case series from Milan, Italy. Transpl Infect Dis. 2020;22(6):e13356.
22. Blanco JL, Ambrosioni J, Garcia F, Martínez E, Soriano A, Mallolas J, et al. COVID-19 in patients with HIV: clinical case series. Lancet HIV. 2020;7(5):e314-e6.
23. Huang J, Lin H, Wu Y, Fang Y, Kumar R, Chen G, et al. COVID-19 in posttransplant patients-report of 2 cases. Am J Transplant. 2020;20(7):1879-81.
24. Pereira MR, Mohan S, Cohen DJ, Husain SA, Dube GK, Ratner LE, et al. COVID-19 in solid organ transplant recipients: Initial report from the US epicenter. Am J Transplant. 2020;20(7):1800-8.
25. de Rojas T, Pérez-Martínez A, Cela E, Baragaño M, Galán V, Mata C, et al. COVID-19 infection in children and adolescents with cancer in Madrid. Pediatr Blood Cancer. 2020;67(7):e28397.
26. Rahimzadeh G, Ekrami Noghabi M, Kadkhodaei Elyaderani F, Navaeifar MR, Enayati AA, Manafi Anari A, et al. COVID-19 Infection in Iranian Children: A Case Series of 9 Patients. JPR. 2020;8(2):139-44.
27. Merza MA, Haleem Al Mezori AA, Mohammed HM, Abdulah DM. COVID-19 outbreak in Iraqi Kurdistan: The first report characterizing epidemiological, clinical, laboratory, and radiological findings of the disease. Diabetes Metab Syndr. 2020;14(4):547-54.
28. Stachel MW, Gidea CG, Reyentovich A, Mehta SA, Moazami N. COVID-19 pneumonia in a dual heart-kidney recipient. J Heart Lung Transplant. 2020;39(6):612-4.

UOB Confidential & Sensitive

29. Righi G, Del Popolo G. COVID-19 tsunami: the first case of a spinal cord injury patient in Italy. Spinal Cord Ser Cases. 2020;6(1):22.
30. Cheng C, Li C, Zhao T, Yue J, Yang F, Yan Y, et al. COVID-19 with rheumatic diseases: a report of 5 cases. Clin Rheumatol. 2020;39(7):2025-9.
31. Poggiali E, Vercelli A, Demichele E, Ioannilli E, Magnacavallo A. Diaphragmatic Rupture and Gastric Perforation in a Patient with COVID-19 Pneumonia. Eur J Case Rep Intern Med. 2020;7(6):001738.
32. Passerini M, Terzi R, Piscaglia M, Passerini S, Piconi S. Disseminated Cryptococcosis in a Patient With Metastatic Prostate Cancer Who Died in the Coronavirus Disease 2019 (COVID-19) Outbreak. Cureus. 2020;12(5):e8254.
33. Holzhauser L, Lourenco L, Sarswat N, Kim G, Chung B, Nguyen AB. Early experience of COVID-19 in 2 heart transplant recipients: Case reports and review of treatment options. Am J Transplant. 2020;20(10):2916-22.
34. Xiong Y, Song S, Ye G, Wang X. Family cluster of three recovered cases of pneumonia due to severe acute respiratory syndrome coronavirus 2 infection. BMJ Case Rep. 2020;13(5).
35. Bastola A, Sah R, Rodriguez-Morales AJ, Lal BK, Jha R, Ojha HC, et al. The first 2019 novel coronavirus case in Nepal. Lancet Infect Dis. 2020;20(3):279-80.
36. Cheng SC, Chang YC, Fan Chiang YL, Chien YC, Cheng M, Yang CH, et al. First case of Coronavirus Disease 2019 (COVID-19) pneumonia in Taiwan. J Formos Med Assoc. 2020;119(3):747-51.
37. Yokoo K, Sugaya F, Matsuzaka S, Ueda K, Kamimura R, Yokoyama T, et al. The first case of COVID-19 occurring as community-acquired pneumonia in Hokkaido, Japan and our preventive measures against nosocomial infection. Respir Med Case Rep. 2020;30:101078.
38. Edrada EM, Lopez EB, Villarama JB, Salva Villarama EP, Dagoc BF, Smith C, et al. First COVID-19 infections in the Philippines: a case report. Trop Med Health. 2020;48:21.
39. Van Cuong L, Giang HTN, Linh LK, Shah J, Van Sy L, Hung TH, et al. The first Vietnamese case of COVID-19 acquired from China. Lancet Infect Dis. 2020;20(4):408-9.
40. Pazgan-Simon M, Rorat M, Buczyńska I, Zińczuk A, Simon K. Gastrointestinal symptoms as the first, atypical indication of severe acute respiratory syndrome coronavirus 2 infection. Pol Arch Intern Med. 2020;130(4):338-9.
41. Caputo V, Schroeder J, Rongioletti F. A generalized purpuric eruption with histopathologic features of leucocytoclastic vasculitis in a patient severely ill with COVID-19. J Eur Acad Dermatol Venereol. 2020;34(10):e579-e81.
42. Baluku JB, Mwebaza S, Ingabire G, Nsereko C, Muwanga M. HIV and SARS-CoV-2 coinfection: A case report from Uganda. J Med Virol. 2020;92(11):2351-3.
43. Jones BA, Slater BJ. Non-operative management of acute appendicitis in a pediatric patient with concomitant COVID-19 infection. J Pediatr Surg Case Rep. 2020;59:101512.
44. Douedi S, Miskoff J. Novel coronavirus 2019 (COVID-19): A case report and review of treatments. Medicine (Baltimore). 2020;99(19):e20207.
45. Kamali Aghdam M, Jafari N, Eftekhari K. Novel coronavirus in a 15-day-old neonate with clinical signs of sepsis, a case report. Infect Dis (Lond). 2020;52(6):427-9.
46. Wang M, Luo L, Bu H, Xia H. One case of coronavirus disease 2019 (COVID-19) in a patient co-infected by HIV with a low CD4(+) T-cell count. Int J Infect Dis. 2020;96:148-50.
47. Meini S, Zini C, Passaleva MT, Frullini A, Fusco F, Carpi R, et al. Pneumatosis intestinalis in COVID-19. BMJ Open Gastroenterol. 2020;7(1).
48. Buonsenso D, Piano A, Raffaelli F, Bonadia N, de Gaetano Donati K, Franceschi F. Point-of-Care Lung Ultrasound findings in novel coronavirus disease-19 pnemoniae: a case report and potential applications during COVID-19 outbreak. Eur Rev Med Pharmacol Sci. 2020;24(5):2776-80.
49. Butt I, Sawlani V, Geberhiwot T. Prolonged confusional state as first manifestation of COVID-19. Ann Clin Transl Neurol. 2020;7(8):1450-2.

50. Tian S, Hu W, Niu L, Liu H, Xu H, Xiao SY. Pulmonary Pathology of Early-Phase 2019 Novel Coronavirus (COVID-19) Pneumonia in Two Patients With Lung Cancer. J Thorac Oncol. 2020;15(5):700-4.
51. Wu Q, Chen T, Zhang H. Recovery from the coronavirus disease-2019 (COVID-19) in two patients with coexisted (HIV) infection. J Med Virol. 2020;92(11):2325-7.
52. Novara E, Molinaro E, Benedetti I, Bonometti R, Lauritano EC, Boverio R. Severe acute dried gangrene in COVID-19 infection: a case report. Eur Rev Med Pharmacol Sci. 2020;24(10):5769-71.
53. Mohan V, Tauseen RA. Spontaneous pneumomediastinum in COVID-19. BMJ Case Rep. 2020;13(5).
54. Suwanwongse K, Shabarek N. Successful Conservative Management of Acute Appendicitis in a Coronavirus Disease 2019 (COVID-19) Patient. Cureus. 2020;12(4):e7834.
55. Millán-Oñate J, Millan W, Mendoza LA, Sánchez CG, Fernandez-Suarez H, Bonilla-Aldana DK, et al. Successful recovery of COVID-19 pneumonia in a patient from Colombia after receiving chloroquine and clarithromycin. Ann Clin Microbiol Antimicrob. 2020;19(1):16.
56. Browne PC, Linfert JB, Perez-Jorge E. Successful Treatment of Preterm Labor in Association with Acute COVID-19 Infection. Am J Perinatol. 2020;37(8):866-8.
57. Zhang H, Xie C, Huang Y. Treatment and Outcome of a Patient With Lung Cancer Infected With Severe Acute Respiratory Syndrome Coronavirus-2. J Thorac Oncol. 2020;15(5):e63-e4.
58. Peng Z, Wang J, Mo Y, Duan W, Xiang G, Yi M, et al. Unlikely SARS-CoV-2 vertical transmission from mother to child: A case report. J Infect Public Health. 2020;13(5):818-20.
59. Cao P, Kong KL, Li F et al., Management of lung cancer patient after surgery with 2019-nCov: A case reort. J. Clin Surg. 2020, 28(2): 195-196.

Observational studies except cohort (1-47)

1. Cao J, Tu WJ, Cheng W, Yu L, Liu YK, Hu X, et al. Clinical Features and Short-term Outcomes of 102 Patients with Coronavirus Disease 2019 in Wuhan, China. Clin Infect Dis. 2020;71(15):748-55.
2. Chen N, Zhou M, Dong X, Qu J, Gong F, Han Y, et al. Epidemiological and clinical characteristics of 99 cases of 2019 novel coronavirus pneumonia in Wuhan, China: a descriptive study. The Lancet. 2020;395(10223):507-13.
3. Du Y, Tu L, Zhu P, Mu M, Wang R, Yang P, et al. Clinical Features of 85 Fatal Cases of COVID-19 from Wuhan. A Retrospective Observational Study. Am J Respir Crit Care Med. 2020;201(11):1372-9.
4. Easom N, Moss P, Barlow G, Samson A, Taynton T, Adams K, et al. Sixty-eight consecutive patients assessed for COVID-19 infection: Experience from a UK Regional infectious diseases Unit. Influenza Other Respir Viruses. 2020;14(4):374-9.
5. Fan Z, Chen L, Li J, Cheng X, Yang J, Tian C, et al. Clinical Features of COVID-19-Related Liver Functional Abnormality. Clin Gastroenterol Hepatol. 2020;18(7):1561-6.
6. Feng Y, Ling Y, Bai T, Xie Y, Huang J, Li J, et al. COVID-19 with Different Severities: A Multicenter Study of Clinical Features. Am J Respir Crit Care Med. 2020;201(11):1380-8.
7. Gautret P, Lagier JC, Parola P, Hoang VT, Meddeb L, Sevestre J, et al. Clinical and microbiological effect of a combination of hydroxychloroquine and azithromycin in 80 COVID-19 patients with at least a six-day follow up: A pilot observational study. Travel Med Infect Dis. 2020;34:101663.
8. Guan W-j, Ni Z-y, Hu Y, Liang W-h, Ou C-q, He J-x, et al. Clinical Characteristics of Coronavirus Disease 2019 in China. New England Journal of Medicine. 2020;382(18):1708-20.
9. Hong KS, Lee KH, Chung JH, Shin KC, Choi EY, Jin HJ, et al. Clinical Features and Outcomes of 98 Patients Hospitalized with SARS-CoV-2 Infection in Daegu, South Korea: A Brief Descriptive Study. Yonsei Med J. 2020;61(5):431-7.
10. Hraiech S, Bourenne J, Kuteifan K, Helms J, Carvelli J, Gainnier M, et al. Lack of viral clearance by the combination of hydroxychloroquine and azithromycin or lopinavir and ritonavir in SARS-CoV-2-related acute respiratory distress syndrome. Ann Intensive Care. 2020;10(1):63.
11. Huang C, Wang Y, Li X, Ren L, Zhao J, Hu Y, et al. Clinical features of patients infected with 2019 novel coronavirus in Wuhan, China. The Lancet. 2020;395(10223):497-506.
12. Ji M, Yuan L, Shen W, Lv J, Li Y, Li M, et al. Characteristics of disease progress in patients with coronavirus disease 2019 in Wuhan, China. Epidemiol Infect. 2020;148:e94-e.
13. Jin X, Lian J-S, Hu J-H, Gao J, Zheng L, Zhang Y-M, et al. Epidemiological, clinical and virological characteristics of 74 cases of coronavirus-infected disease 2019 (COVID-19) with gastrointestinal symptoms. Gut. 2020;69(6):1002.
14. Lian J, Jin X, Hao S, Jia H, Cai H, Zhang X, et al. Epidemiological, clinical, and virological characteristics of 465 hospitalized cases of coronavirus disease 2019 (COVID-19) from Zhejiang province in China. Influenza Other Respir Viruses. 2020;14(5):564-74.
15. Lin L, Jiang X, Zhang Z, Huang S, Zhang Z, Fang Z, et al. Gastrointestinal symptoms of 95 cases with SARS-CoV-2 infection. Gut. 2020;69(6):997.
16. Liu BM, Yang QQ, Zhao LY, Xie W, Si XY. Epidemiological characteristics of COVID-19 patients in convalescence period. Epidemiol Infect. 2020;148:e108.
17. Mahévas M, Tran V-T, Roumier M, Chabrol A, Paule R, Guillaud C, et al. Clinical efficacy of hydroxychloroquine in patients with covid-19 pneumonia who require oxygen: observational comparative study using routine care data. BMJ. 2020;369:m1844.

18. Maraj I, Hummel JP, Taoutel R, Chamoun R, Workman V, Li C, et al. Incidence and determinants of QT interval prolongation in COVID-19 patients treated with hydroxychloroquine and azithromycin. J Cardiovasc Electrophysiol. 2020;31(8):1904-7.
19. Mathian A, Mahevas M, Rohmer J, Roumier M, Cohen-Aubart F, Amador-Borrero B, et al. Clinical course of coronavirus disease 2019 (COVID-19) in a series of 17 patients with systemic lupus erythematosus under long-term treatment with hydroxychloroquine. Annals of the Rheumatic Diseases. 2020;79(6):837.
20. Pan L, Mu M, Yang P, Sun Y, Wang R, Yan J, et al. Clinical Characteristics of COVID-19 Patients With Digestive Symptoms in Hubei, China: A Descriptive, Cross-Sectional, Multicenter Study. Am J Gastroenterol. 2020;115(5):766-73.
21. Peng H, Gao P, Xu Q, Liu M, Peng J, Wang Y, et al. Coronavirus disease 2019 in children: Characteristics, antimicrobial treatment, and outcomes. J Clin Virol. 2020;128:104425.
22. Pereira A, Cruz-Melguizo S, Adrien M, Fuentes L, Marin E, Perez-Medina T. Clinical course of coronavirus disease-2019 in pregnancy. Acta Obstetricia et Gynecologica Scandinavica. 2020;99(7):839-47.
23. Piva S, Filippini M, Turla F, Cattaneo S, Margola A, De Fulviis S, et al. Clinical presentation and initial management critically ill patients with severe acute respiratory syndrome coronavirus 2 (SARS-CoV-2) infection in Brescia, Italy. J Crit Care. 2020;58:29-33.
24. Sandhu A, Tillotson G, Polistico J, Salimnia H, Cranis M, Moshos J, et al. Clostridioides difficile in COVID-19 Patients, Detroit, Michigan, USA, March-April 2020. Emerg Infect Dis. 2020;26(9):2272-4.
25. Shah SJ, Barish PN, Prasad PA, Kistler A, Neff N, Kamm J, et al. Clinical features, diagnostics, and outcomes of patients presenting with acute respiratory illness: A retrospective cohort study of patients with and without COVID-19. EClinicalMedicine. 2020;27:100518.
26. Soltani J, Sedighi I, Shalchi Z, Sami G, Moradveisi B, Nahidi S. Pediatric coronavirus disease 2019 (COVID-19): An insight from west of Iran. North Clin Istanb. 2020;7(3):284-91.
27. Sun L, Shen L, Fan J, Gu F, Hu M, An Y, et al. Clinical features of patients with coronavirus disease 2019 from a designated hospital in Beijing, China. J Med Virol. 2020;92(10):2055-66.
28. Wang D, Hu B, Hu C, Zhu F, Liu X, Zhang J, et al. Clinical Characteristics of 138 Hospitalized Patients With 2019 Novel Coronavirus–Infected Pneumonia in Wuhan, China. JAMA. 2020;323(11):1061-9.
29. Wang L, Duan Y, Zhang W, Liang J, Xu J, Zhang Y, et al. Epidemiologic and Clinical Characteristics of 26 Cases of COVID-19 Arising from Patient-to-Patient Transmission in Liaocheng, China. Clin Epidemiol. 2020;12:387-91.
30. Wang R, Pan M, Zhang X, Han M, Fan X, Zhao F, et al. Epidemiological and clinical features of 125 Hospitalized Patients with COVID-19 in Fuyang, Anhui, China. Int J Infect Dis. 2020;95:421-8.
31. Wang Z, Yang B, Li Q, Wen L, Zhang R. Clinical Features of 69 Cases With Coronavirus Disease 2019 in Wuhan, China. Clinical Infectious Diseases. 2020;71(15):769-77.
32. Wu H, Zhu H, Yuan C, Yao C, Luo W, Shen X, et al. Clinical and Immune Features of Hospitalized Pediatric Patients With Coronavirus Disease 2019 (COVID-19) in Wuhan, China. JAMA Network Open. 2020;3(6):e2010895-e.
33. Yang L, Liu J, Zhang R, Li M, Li Z, Zhou X, et al. Epidemiological and clinical features of 200 hospitalized patients with corona virus disease 2019 outside Wuhan, China: A descriptive study. Journal of clinical virology : the official publication of the Pan American Society for Clinical Virology. 2020;129:104475-.
34. Yang Q, Xie L, Zhang W, Zhao L, Wu H, Jiang J, et al. Analysis of the clinical characteristics, drug treatments and prognoses of 136 patients with coronavirus disease 2019. Journal of Clinical Pharmacy and Therapeutics. 2020;45(4):609-16.
35. Ye G, Pan Z, Pan Y, Deng Q, Chen L, Li J, et al. Clinical characteristics of severe acute respiratory syndrome coronavirus 2 reactivation. J Infect. 2020;80(5):e14-e7.
36. Yu B, Li C, Chen P, Zhou N, Wang L, Li J, et al. Low dose of hydroxychloroquine reduces fatality of critically ill patients with COVID-19. Sci China Life Sci. 2020;63(10):1515-21.

37. Yu N, Li W, Kang Q, Xiong Z, Wang S, Lin X, et al. Clinical features and obstetric and neonatal outcomes of pregnant patients with COVID-19 in Wuhan, China: a retrospective, single-centre, descriptive study. The Lancet Infectious Diseases. 2020;20(5):559-64.
38. Yu Y, Xu D, Fu S, Zhang J, Yang X, Xu L, et al. Patients with COVID-19 in 19 ICUs in Wuhan, China: a cross-sectional study. Critical Care. 2020;24(1):219.
39. Zhang J, Yu M, Tong S, Liu LY, Tang LV. Predictive factors for disease progression in hospitalized patients with coronavirus disease 2019 in Wuhan, China. J Clin Virol. 2020;127:104392.
40. Zheng Y, Sun LJ, Xu M, Pan J, Zhang YT, Fang XL, et al. Clinical characteristics of 34 COVID-19 patients admitted to intensive care unit in Hangzhou, China. J Zhejiang Univ Sci B. 2020;21(5):378-87.
41. Zhong Q, Liu YY, Luo Q, Zou YF, Jiang HX, Li H, et al. Spinal anaesthesia for patients with coronavirus disease 2019 and possible transmission rates in anaesthetists: retrospective, single-centre, observational cohort study. Br J Anaesth. 2020;124(6):670-5.
42. Yu N, Fang ZX, Wu JL, et al. Novel coronavirus pneumonia in pregancy: perinatal outcomes. Progress in obstetrics and gynecology. 2020. 3:167-9.
43. Zhou SY, Wang CT, Zhang W, et al. Clinical characteristics and treatment effect of 537 cases of novel coronavirus pneumonia in Shandong Province. Journal of Shandong University (Health Sciences). 2020; 58(3): 44-51.
44. Yang J, Jie CY, Yang CY. Common type of COVID-19: Clinical analysis of 40 cases. Practical Journal of Cardiac Cerebral Pneumal and Vascular Disease. 2020; 28(2): 1-4.
45. Li YL, Shan NB, Sun W, et al. Comparative study for clinical features between COVID-19 patients with conventionaltype and heavy/critical type. Practical Journal of Cardiac Cerebral Pneumal and Vascular Disease. 2020; 28(3): 14-8.
46. Lv RB, Wang WJ, Li X. Clinical Observation on *Lianhua Qingwen Granules* combined with western medicine conventional therapy in the treatment of 63 suspected cases of Coronavirus disease 2019. Journal of Traditional Chinese Medicine. 2020; 61(8): 655-9.
47. Gautret P, Lagier JC, Parola P, Hoang VT, Meddeb L, Mailhe M, et al. Hydroxychloroquine and azithromycin as a treatment of COVID-19: results of an open-label non-randomized clinical trial. Int J Antimicrob Agents. 2020;56(1):105949.

UOB Confidential & Sensitive

Cohort and RCT (1-12)

1. Borba MGS, Val FFA, Sampaio VS, Alexandre MAA, Melo GC, Brito M, et al. Effect of High vs Low Doses of Chloroquine Diphosphate as Adjunctive Therapy for Patients Hospitalized With Severe Acute Respiratory Syndrome Coronavirus 2 (SARS-CoV-2) Infection: A Randomized Clinical Trial. JAMA Netw Open. 2020;3(4):e208857.

2. Cao B, Wang Y, Wen D, Liu W, Wang J, Fan G, et al. A Trial of Lopinavir-Ritonavir in Adults Hospitalized with Severe Covid-19. N Engl J Med. 2020;382(19):1787-99.

3. Chen Z, Hu J, Zhang Z, Jiang S, Han S, Yan D, et al. Efficacy of hydroxychloroquine in patients with COVID-19: results of a randomized clinical trial. medRxiv. 2020:2020.03.22.20040758.

4. Cheng Y, Luo R, Wang K, Zhang M, Wang Z, Dong L, et al. Kidney disease is associated with in-hospital death of patients with COVID-19. Kidney Int. 2020;97(5):829-38.

5. Chorin E, Dai M, Shulman E, Wadhwani L, Bar-Cohen R, Barbhaiya C, et al. The QT interval in patients with COVID-19 treated with hydroxychloroquine and azithromycin. Nat Med. 2020;26(6):808-9.

6. Hung IF-N, Lung K-C, Tso EY-K, Liu R, Chung TW-H, Chu M-Y, et al. Triple combination of interferon beta-1b, lopinavir–ritonavir, and ribavirin in the treatment of patients admitted to hospital with COVID-19: an open-label, randomised, phase 2 trial. The Lancet. 2020;395(10238):1695-704.

7. Mercuro NJ, Yen CF, Shim DJ, Maher TR, McCoy CM, Zimetbaum PJ, et al. Risk of QT Interval Prolongation Associated With Use of Hydroxychloroquine With or Without Concomitant Azithromycin Among Hospitalized Patients Testing Positive for Coronavirus Disease 2019 (COVID-19). JAMA Cardiol. 2020;5(9):1036-41.

8. Tang W, Cao Z, Han M, Wang Z, Chen J, Sun W, et al. Hydroxychloroquine in patients with mainly mild to moderate coronavirus disease 2019: open label, randomised controlled trial. Bmj. 2020;369:m1849.

9. Wang Y, Zhang D, Du G, Du R, Zhao J, Jin Y, et al. Remdesivir in adults with severe COVID-19: a randomised, double-blind, placebo-controlled, multicentre trial. Lancet. 2020;395(10236):1569-78.

10. Ye XT, Luo YL, Xia SC, Sun QF, Ding JG, Zhou Y, et al. Clinical efficacy of lopinavir/ritonavir in the treatment of Coronavirus disease 2019. Eur Rev Med Pharmacol Sci. 2020;24(6):3390-6.

11. Rosenberg ES, Dufort EM, Udo T, Wilberschied LA, Kumar J, Tesoriero J, et al. Association of Treatment With Hydroxychloroquine or Azithromycin With In-Hospital Mortality in Patients With COVID-19 in New York State. Jama. 2020;323(24):2493-502.

12. Zhang Y, Cui Y, Shen M, Zhang J, Liu B, Dai M, et al. Association of diabetes mellitus with disease severity and prognosis in COVID-19: A retrospective cohort study. Diabetes Res Clin Pract. 2020;165:108227.

UOB Confidential & Sensitive
